# Access and adequacy of antenatal care in a city in Brazil during two phases of the COVID-19 pandemic

**DOI:** 10.61622/rbgo/2024rbgo87

**Published:** 2024-10-23

**Authors:** Nicole Zazula Beatrici, Roxana Knobel, Mariana Schmidt Vieira, Iago Felipe Alexandrini, Alberto Trapani, Carla Betina Andreucci

**Affiliations:** 1 Universidade Federal de Santa Catarina Florianópolis SC Brazil Universidade Federal de Santa Catarina, Florianópolis, SC, Brazil.; 2 Universidade Federal de São Carlos São Carlos São Paulo Brazil Universidade Federal de São Carlos, São Carlos, São Paulo, Brazil.

**Keywords:** Prenatal care, Pregnancy, Postpartum period, COVID-19, Pandemics, Vaccination, Diabetes, gestational, Delivery of health care, Surveys and questionnaires

## Abstract

**Objective:**

To compare access and suitability of antenatal care between years 2020 and 2022 among postpartum individuals at a Hospital in Florianopolis, and evaluate factors associated with antenatal suitability.

**Methods:**

Observational, cross-sectional, and quantitative study carried out in 2022. Collected data were compared with the database of a previous similar study carried out in the same setting in 2020. Data were extracted from medical records and prenatal booklets, in addition to a face-to-face questionnaire. Adequacy was measured using the Carvalho and Novaes index and health access was qualitatively evaluated. Socio-demographic and antenatal variables were analyzed. A statistical significance level of 0.05 was considered. Open-ended questions were categorized for analysis.

**Results:**

395 postpartum individuals were included. Antenatal care was adequate for 48.6% in 2020 and 69.1% in 2022. Among the barriers to access, 56% reported difficulty in scheduling appointments and/or exams and 23% complained of reduced healthcare staff due to strikes, COVID-19, among others. Adequate antenatal care was associated with being pregnant in 2022, being referred to high-risk units (PNAR), and not reporting difficulties in access. Also, it was associated with twice the chance of investigation for gestational diabetes (GDM) and syphilis.

**Conclusion:**

The 2022 post-vaccination period showed higher antenatal adequacy. The main difficulty for postpartum individuals was scheduling appointments and/or exams. Having antenatal care in 2022, no reports of difficulty in access, and follow-up at a high-risk unit were associated with antenatal adequacy.

## Introduction

Adequate antenatal care directly impacts maternal and neonatal morbidity and mortality. It is also a guaranteed right for every person in Brazil, available universally and free of charge through the Brazilian Unified Health System.^(1)^ Antenatal care provides opportunities for stablishing strong bonds between health teams and the pregnant individuals, guidance to the gestational process, recognition of clinical warning signs, encouragement for a vaginal birth and breastfeeding, and immunization, among further benefits.^(1,2)^ However, qualified antenatal care encompasses more than just checking in at regular appointments. It is paramount to request and interpret exams, as well as ensure rapid transport to specialized centers if necessary.^(1)^

Quantifiable parameters for evaluating the suitability of antenatal care have been systematically proposed.^(3–5)^ The Carvalho and Novaes index applies two variables to assess the adequacy of care, namely the gestational age at the beginning of the clinical follow-up and the total number of appointments.^(4)^ The difference between the indexes is the cutoff points for adequacy, considering the gestational age at 12, 14, 16, and up to 20 weeks, and the number of visits ranging from six to nine, or at least 80 % of expected scheduled consultations.^(3–5)^

The COVID-19 pandemic seriously affected antenatal adequacy. There were difficulties with health care access, restraints for moving around due to social isolation, fear of contagion at health facilities, shortage of health care providers, and the care of respiratory symptomatic patients was prioritized over healthy subjects. Therefore, antenatal care indicators worsened during this period in Brazil.^(2,6)^

The development of COVID-19 vaccines allowed the immunization of most of the population, and social isolation began to be recommended only for symptomatic cases.^(7,8)^ Hence, after the extensive availability of immunization and the complete resumption of primary health care assistance, an improvement in the accessibility and quality of obstetric care was expected.

The present study was carried out with postpartum individuals at the University Hospital of Florianopolis. The suitability and difficulties in accessing antenatal care during the peak of the health emergency of the COVID-19 pandemic in 2020 were compared with the year 2022, after extensive immunization. Additionally, we analysed factors associated with antenatal suitability in a southern capital city in Brazil.

## Methods

Both were cross-sectional studies with records from medical charts and prenatal booklets, as well as data from a questionnaire applied to postpartum individuals admitted at the University Hospital (HU) Polydoro Ernani de São Thiago at the Federal University of Santa Catarina (UFSC) in the city of Florianopolis, Brazil. The Research Hospital, a tertiary care facility and a reference center, provides care for pregnancies of both high and low risk. We followed the STROBE initiative.^(9)^

We calculated the sample size with a population parameter estimate, 95% CI, margin of error of 5%, and 17.4% of expected inadequate antenatal care among subjects.^(2)^ A total sample of 221 individuals would be necessary to evaluate antenatal quality of care.

Postpartum individuals who gave birth to live babies between 35 and 42 gestation weeks during the COVID-19 pandemic at the chosen hospital were eligible for the study. Individuals were excluded if they were under 18 years of age, referred to high-risk antenatal care (PNAR) at the first trimester of pregnancy, carried babies with malformations, were diagnosed with mental disorders, were foreigners without fluency in Portuguese or who arrived in Brazil after the first trimester of pregnancy.

A standard confidential self-completion questionnaire with open and closed questions was applied. The instrument had already been tested,^(2)^ and we included the question about COVID-19 vaccination at the 2022 interview. Information about antenatal care was obtained from pregnancy booklets and hospital records. Data were anonymized and plotted at a database specially created for the study. The primary outcomes were suitability of antenatal care and difficulties for accessing antenatal care.

Adequacy was measured by the Carvalho and Novaes index: antenatal care was considered adequate if pregnancy follow-up started before or during the 13th gestational week, as well as if seven or more clinical appointments were completed.^(10)^ Access was analysed based on the difficulties reported by individuals during the pregnancy follow-up.

We analyzed socioeconomic characteristics and antenatal goals, as well as health insurance (public or private), reference to high-risk units, and reports of barriers to access health care. Antenatal goals were screening for gestational diabetes (GDM), HIV, syphilis and toxoplasmosis, adequate weight gain, and receiving the COVID-19 vaccine. We considered antenatal care adequate if documented GDM screening with fasting blood glucose during the first and second half of the pregnancy, or oral glucose tolerance test after 24 weeks gestation, Syphilis and HIV testing at 1st and 3rd trimesters, toxoplasmosis testing quarterly for susceptible patients or at least once for previously immunized individuals,^(11)^ not exceeding expected weight gain according to the Brazilian Ministry of Health,^(11)^ and receiving at least one dose of the COVID-19 vaccine.

We applied SPSS 27 statistical program for analysis, with 0.05 of statistical significance level, chi square or Fisher's exact test, Student's T test and relative risk calculation. We carried out two multivariate analyses using binary logistic regression with the Forward LR method, one with socio-demographic variables and antenatal characteristics, and another with variables related to antenatal care. The variables that showed significance in the crude analysis were included in the model. The models were corrected for potentially confounding variables (cohabiting with a partner, age, attendance at PNAR, non-white race, and year inclusion in the study). The open questions allowed textual responses, which were divided into categories and analyzed quantitatively to identify patterns and trends. This involved developing a coding system, ensuring intercoder reliability, and conducting descriptive statistical analyses to summarize the findings.

We compared data from the study "Antenatal care received by postpartum women at the Florianopolis University Hospital during the COVID-19 Pandemic", number CAAE 52681221.7.0000.0121, conducted between March and May 2022, with the database from the study "Obstetric and puerperal complications during the COVID-19 pandemic", number CAAE 5543120.7.0000.0121, conducted from October to December 2020 at the same setting (data already published).^(2)^

## Results

Six hundred and ninety-seven individuals met the selection criteria at both periods. 198 were excluded due to refusal (68), unavailable (sleeping, bathing, or in respiratory isolation), or unable to complete the questionnaire (130). 104 cases were excluded due to fetal death, fetal malformation, below 35 gestation weeks, or lack of necessary documented variables. The final sample was 395 postpartum individuals, 175 from the year 2020 and 220 from the year 2022. Most individuals were multiparous (57.7%), between 20 and 35 years of age (73.9%), and self-declared white (56.2%). Approximately 62% had completed secondary education, and 41.7% had formal employment. 46.7% reported a monthly income of up to two Minimum Brazilian Wage (U$500.00). 52.1% were overweight or obese before pregnancy. 83.3% were attended through the Brazilian Public Health System (SUS) (Table 1).

**Table 1:** Sociodemographic, clinical, and antenatal care characteristics

Variables		2020 n(%)	2022 n(%)	Total n(%)
Primiparous		81(46,3)	86(39,1)	167(42,3)
Age (years)				
	≤ 20	19(10,9)	17(7,7)	36(9,1)
	20-35	118(67,4)	163(74,1)	281(71,1)
	> 35	38(21,7)	40(18,2)	78(36)
Skin colour (n=393)				
	Asiatic	6(3,5)	2(0,9)	8(2)
	White	100(57,8)	121(55)	221(56,2)
	Brown	40(23,1)	67(30,5)	107(27,2)
	Black	24(13,9)	30(13,6)	54(13,7)
	Indigenous	3(1,7)	0(0)	3(0,8)
Level of education (n=393)				
	None	15(8,6)	5(2,3)	20(5,1)
	Primary school	21(12)	26(11,8)	47(11,9)
	Secondary school	92(52,6)	150(68,2)	242(61,3)
	Undergraduate degree	45(25,7)	39(17,7)	84(21,3)
Employment (n=386)				
	Formal	79(48,5)	84(38,5)	163(42,8)
	Informal	33(20,2)	37(17)	70(18,4)
	Not working	51(31,3)	97(44,5)	148(38,8)
Up to U$ 500.00 monthly income (n=390)		75(43,4)	107(49,3)	182(46,7)
BMI* prior to current gestation (n=367)				
	Adequate or Underweight	78(49,7)	99(47,1)	177(48,2)
	Overweight	49(31,2)	59(28,1)	108(29,4)
	Obesity	30(19,1)	52(24,8)	82(22,3)
Living with partner (n=384)		137(82,5)	188(86,2)	325(84,6)
Health insurance				
	Public	141(80,6)	188(85,5)	329(83,3)
	Private	14(8)	12(5,5)	26(6,6)
	Combined public and private	20(11,4)	20(9,1)	40(10,1)

* BMI - Body Mass Index

In average, antenatal follow-up started at 12 weeks and had seven appointments completed, and at 9 weeks with eight appointments during 2020 and 2022, respectively. Antenatal care was significantly more adequate in 2022 than in 2020 (OR=2.36, 95%CI 1.56-3.57). The percentage of people reporting barriers to healthcare access was 49.1% in 2020 and 42.3% in 2022 (not significant). There were few remote appointments in 2022 (OR=0.127, 95%CI 0.077-0.208) (Table 2). Through open-ended questions about access to healthcare, we identified and categorized 119 items into eight response categories. The majority of responses (56%) were related to scheduling appointments and/or exams, while 12% of responses mentioned a shortage of healthcare professionals due to strikes or absences, and 11% attributed difficulties to the COVID-19 pandemic. Ten patients (8%) reported a poor relationship with the healthcare team and 5% faced difficulties in transporting them to appointments. For 3% of those interviewed, the health establishment was overcrowded and another 3% cited personal reasons for missing appointments, such as caring for children or siblings, not being able to miss work, among others. A small fraction of patients (1%) reported long intervals between appointments (data not shown).

**Table 2:** Comparison between the years 2020 and 2022 regarding antenatal suitability, barriers to access and remote appointments

	Year			
Variables	2020 Median (SD)	2022 Median (SD)	OR [CI95%]	p-value
Number of appointments	7.09(2.77)	8.45(2.92)	1.18 * (1.1-1.27)	<0.001
Gestational age at beginning of follow-up	12.35(7.27)	9.58(5.62)	0.93 * (0.90-0.96)	<0.001
Variables	2020 n(%)	2022 n(%)	OR [CI95%]	p-value
Adequate (≤ 13 weeks and ≥ 7 appointments)	85(48.6)	152(69.1)	2.36 (1.56-3.57)	<0.001
Reported barriers to health access	85(49.1)	93(42.3)	0.75 (0.5-1.13)	0.175
Had remote appointment	91(54.5)	29(13.2)	0.127 (0.077-0.208)	<0.001

OR - odds ratio; SD=standard deviation; *Exp (B) - exponentiated coefficient Adjusted analysis for confounding factors (age, social class, and isolation during the COVID-19 pandemic); Hosmer-Lemeshow - 0.494

The suitability of care according to socioeconomic data and antenatal follow-up revealed that having attendance during 2022, being referred to high-risk units, not reporting barriers to access, living with partners and/or not having remote appointments were associated with a greater chance of adequate antenatal care (the last two variables showed no significance after multivariate analysis) (Table 3). The binary logistic regression model for the significant sociodemographic data (p<0.05) considering the variable of non-adequate antenatal showed significance for the variables difficulty for access (p<0.001; OR 2.33; 95%CI 1.5-3.6), care at high-risk unit (PNAR) (p=0.002; OR 0.4; 95%CI 0.22-0.72) and the year 2020 (p<0.001; OR 2.41; CI95 % 1.55-3.74); with a Hosmer Lemeshow Test significance of 0.93 (Table 3). At the binary logistic regression to evaluate the influence of adequate antenatal care, the complete investigation for GDM and syphilis remained significant (Table 3). All participants had at least one HIV test (data not shown in tables).

**Table 3:** Risk of Inadequate Prenatal Care: Odds Ratios and Confidence Intervals across sociodemographic and antenatal variables

Sociodemographic variables and antenatal variables	Adequate n(%)	Inadequate n(%)	p-value	Crude OR (CI 95%)	Adjusted OR**(CI 95%)
Maternal age					
<20	18(7.6)	18(11.4)	0.23	1.53 (0.76-3.07)	
21-34	170(71.7)	111(70.3)	1	1	
35 or older	49(20.7)	29(18.4)	0.72	0.91 (0.54-1.52)	
Not white	95(40.1)	77(49.4)	0.07	1.46 (0.97-2.19)	
Secondary school	200(84.7)	126(80.3)	0.25	0.73 (0.43-1.24)	
Paid Employment	152(65)	91(59.9)	0.3	0.8 (0.53-1.23)	
Familiar income< 2 US$ 500.00	107(45.5)	75(48.4)	0.58	0.89 (0.59-1.34)	
Living with partner	203(88.3)	122(79.2)	0.02	0.51 (0.29-0.89)	0.57 (0.31-1.04)
Primiparous	105(44.3)	62(39.2)	0.3	1.23 (0.82-1.85)	
Barrier for health access reported	88(37.1)	90(50.6)	<0.001	2.31 (1.58-3.49)	2.25 (1.45-3.49)
Year 2020	85(35.9)	90(57)	<0.001	2.36 (1.57-3.57)	1.95 (1.19-3.19)
At least one private appointment*	34(14.3)	32(20.3)	0.12	1.51 (0.89-2.58)	
High-risk unit PNAR	62(26.2)	20(12.7)	0.001	0.41 (0.23-0.71)	0.43 (0.23-0.78)
Remote appointments	56(24)	64(41.6)	<0.001	2.24 (1.45-3.49)	1.65 (0.98-2.77)
		Antenatal variables			
Complete Syphilis screening	191(80.6)	90(57)	< 0.001	0.32 (0.2-0.5)	0.5 (0.29-0.86)
Complete Toxoplasmosis screening	177(74.7)	89(56.3)	< 0.001	0.44 (0.28-0.67)	0.72 (0.42-1.23)
Complete GDM screening	171(72.2)	77(48.7)	< 0.001	0.37 (0.24-0.56)	0.5 (0.31-0.81)
COVID-19 immunization	119(50.2)	57(36.1)	0.006	0.56 (0.37-0.84)	0.71 (0.31-1.62)
High weight gain	102(43.2)	39(28.9)	0.006	0.53 (0.34-0.84)	0.66 (0.42-1.07)

* 26 (6.6%) participants had private care exclusively;

** Quality of adjustment H&L=0.93 for sociodemographic and antenatal variables, and 0.41 for antenatal variables corrected by year, barrier to health access, and attendance at high-risk unit

## Discussion

During 2020, in the COVID-19 pandemic context, before vaccines were available, pregnant individuals started antenatal follow-up later and had fewer face-to-face appointments, as well as more frequent remote consultations. As a result, antenatal care adequacy rates were significantly better in 2022. Our data is in line with findings from India^(12)^ and from the United Kingdom^(13)^ at the same period.

The COVID-19 pandemic struck the world with high daily morbidity and mortality rates, requiring the reorganization of the healthcare system to accommodate the growing demand for infected patients.^(6,14)^ Furthermore, special concern was evolved towards pregnant and postpartum individuals, as they showed greater chances of severe illness and death, in addition to obstetric complications such as preterm birth and pre-eclampsia.^(6,14–17)^

Social isolation was necessary to reduce the risk of contagion yet made access to antenatal care difficult.^(18)^ Availability of family planning programs and legal abortion were also impaired.^(12)^ To minimize the impact generated by the reduction of face-to-face consultation, several countries managed to maintain health care using software, remote appointments, and drive-through attendance.^(6,12,14)^ In our sample, remote consultations were used by more than half of individuals in 2020. Telemedicine was an alternative to reduce the exposure to public transport and health units, and to alleviate the anxiety caused by the lack of access to healthcare professionals during a vulnerable time.^(6)^

The real effectiveness of online appointments is questionable in developing countries, where people do not have access to the necessary technology, especially in rural areas.^(12)^ In the obstetric context, the impossibility of physical examination such as blood pressure and fundal height measurement, as well as fetal heartbeat auscultation may lead to worse maternal and perinatal health outcomes.^(6,12,14)^

Florianopolis is the capital of Santa Catarina State and has 100% coverage by the Family Health Program (PSF), with prenatal adequacy rates at 77% in 2019.^(19)^ Nevertheless, difficulties in accessing primary health care were reported before the COVID-19 pandemic.^(20)^ Contributing to this scenario were undersized/underfunded primary care, excessive number of individuals and reduced staff within health teams, bureaucracy, and functional issues at health units.^(20)^ In our study, the main difficulties reported by pregnant individuals were delays in scheduling exams and appointments, as described by the Indian population.^(12)^ Differently, findings from Northeastern Brazil showed difficulties related to the lack of public transport and delays in scheduling laboratory and ultrasound exams, with fewer reports on problems with scheduling appointments.^(18)^

As expected, pregnant individuals who faced barriers to accessing health care had delays in starting clinical follow-up and fewer consultations, directly impacting the suitability index. Antenatal adequacy was greater among patients who underwent antenatal care in 2022, who did not report access difficulties or were referred to high-risk units (PNAR). High-risk conditions led to more frequent clinical appointments and complementary tests, hence increasing the suitability index.^(11,21)^ Access to PNAR for complicated pregnancies varies according to regional configurations and public health networks,^(22)^ not available to be assessed for analysis in our study.

Quality of care encompasses health professional training, timely request of exams, proper diagnosis of threatening conditions, as well as adequate patient treatment and follow-up.^(4,5)^ The suitability of antenatal care measurement is challenging, requiring quantifiable factors to be compared. Several suitability indexes have been proposed, although non-uniformity between instruments makes comparison between studies and different populations troublesome.^(4)^ We applied the Carvalho and Novaes quantitative index, which presents agreement and high accuracy (approximately 80%) when compared to other instruments,^(4)^ in addition to being simple and applicable for live birth registry data.

In our study, individuals whose antenatal care met the goal criteria were twice as likely to have been fully investigated for syphilis and GDM. For both conditions, timely diagnosis and treatment can modify the prognosis for mother and baby.^(23–25)^ Maternal hyperglycemia increases risks of miscarriage, pre-eclampsia, fetal macrosomia, and birth injuries, in addition to cardiovascular disease, overweight, and type 2 diabetes for both mother and child.^(23)^ In Brazil, maternal syphilis increased sharply from 2010 to 2019 (3.5 to 20.8 cases per 1,000 live births) and it is associated with miscarriage, stillbirth, preterm birth, and congenital syphilis (1.4 to 8.2 cases per 1,000 live births in the same period).^(11)^ The earlier the diagnosis and management, the better the maternal-fetal prognosis.^(11,25)^

Additional remarks in our findings can be described. In 2022, several individuals received assistance below adequate, despite the improvement compared to the year 2020. Reported difficulties in accessing health were directly related to the inadequacy of care and could be minimized by facilitating the scheduling and the completing of exams, public transport to remote areas, as well as full and operating health teams.^(20)^

Noteworthy, a reduction in the diagnosis of syphilis and diabetes due to inadequate antenatal care was observed using the Carvalho and Novaes index quantitative tool.^(11)^ It is crucial to tackle the issues despite the structural limitations that affect Brazilian SUS. Thus, strategies like the active search for individuals at the beginning of pregnancy, an alert system for patients who have not undergone basic exams and/or prescribed treatment, as well as continued team training should be implemented.^(3,20)^

Our study limitations are the sample size, as well as a possible selection bias due to the studied population (pregnant individuals from a large southern capital). It was not possible to determine if included individuals had more years of schooling or higher socioeconomic status than the average population and, therefore may have faced fewer barriers to access health care. Further, the suitability of antenatal care was measured quantitatively only, a difficulty already described elsewhere.^(4)^

Our strength was to analyse the barriers to healthcare access and the extent to which antenatal adequacy can be associated with the investigation of conditions that affect maternal and fetal health. Such publications are scarce, and we did not find studies comparing results during and after the COVID-19 pandemic.

## Conclusion

In 2020, 48.6% of antenatal care was assessed as adequate, while this increased to 69.1% in 2022. Inadequate antenatal care was significantly associated with pregnancy in 2020, non-referral to high-risk prenatal care units (PNAR) and reported challenges in access. Conversely, adequate prenatal care was linked to a two-fold higher likelihood of screening for gestational diabetes mellitus (GDM) and syphilis.
